# Supplementary material for: Polyketide synthase gene clusters in the ericoid mycorrhizal fungus Oidiodendron maius: genome-wide prediction, expression profiling, and functional characterization of knockout mutants
Source: Front Plant Sci. 2026 Sep 16;17:1924430. doi: 10.3389/fpls.2026.1924430 (PMC13623903; doi:10.3389/fpls.2026.1924430)
Supplement: Supplementary file 1 [file SupplementaryFile1.zip › Supplementary Materials/Supplementary Folder 1.zip!/Oidma1_AssemblyScaffolds_Repeatmasked/knownclusterblast/cluster7/jgi.p_Oidma1_108429_mibig_hits.html]

| MIBiG Protein | Description | MIBiG Cluster | MiBiG Product | % ID | % Coverage | BLAST Score | E-value |
| --- | --- | --- | --- | --- | --- | --- | --- |
| ABD65949.1 | hydroxyphenylglycine\_aminotransferase/hydroxymandelate\_oxidase\_fusion\_protein | BGC0000341 | Enduracidin\_biosynthetic\_gene\_cluster | 37 | 76.1388286334 | 213 | 5.1e-55 |
| AGS77333.1 | Hmo | BGC0001178 | UK-68,597\_biosynthetic\_gene\_cluster | 37 | 75.9219088937 | 210 | 5.6e-54 |
| ALV82386.1 | putative\_oxidase | BGC0001370 | Lipopeptide\_8D1-1\_/\_lipopeptide\_8D1-2\_biosynthetic\_gene\_cluster | 37 | 78.7418655098 | 209 | 9.6e-54 |
| AAK81834.1 | glycolate\_oxidase | BGC0000326 | Complestatin\_biosynthetic\_gene\_cluster | 36 | 77.0065075922 | 208 | 1.6e-53 |
| CAB38520.1 | putative\_glycolate\_oxidase | BGC0000315 | Calcium-dependent\_antibiotic\_biosynthetic\_gene\_cluster | 37 | 76.1388286334 | 207 | 3.6e-53 |
| AAM80552.1 | Hmo | BGC0000290 | A47934\_biosynthetic\_gene\_cluster | 36 | 75.704989154 | 202 | 1.2e-51 |
| EWG54275.1 | hypothetical\_protein | BGC0001190 | Fusaric\_acid\_biosynthetic\_gene\_cluster | 35 | 70.715835141 | 183 | 7.3e-46 |
| ALK27900.1 | hydroxymandelate\_oxidase | BGC0001233 | Feglymycin\_biosynthetic\_gene\_cluster | 35 | 78.7418655098 | 196 | 4.9e-50 |
| AIG79221.1 | Hypothetical\_protein | BGC0000419 | Ristomycin\_A\_biosynthetic\_gene\_cluster | 36 | 75.9219088937 | 193 | 4.2e-49 |
| AIE77079.1 | putative\_phenylglycolate\_oxidase | BGC0000418 | Ristocetin\_biosynthetic\_gene\_cluster | 36 | 76.3557483731 | 193 | 4.2e-49 |
| CAD91196.1 | putative\_hydroxymandelate\_oxidase | BGC0000289 | A40926\_biosynthetic\_gene\_cluster | 35 | 76.1388286334 | 193 | 5.4e-49 |
| sln2 | 2-hydroxy-acid\_oxidase | BGC0001230 | Salinamides\_biosynthetic\_gene\_cluster | 34 | 76.5726681128 | 190 | 4.6e-48 |
| AAT09795.1 | NocN | BGC0000395 | Nocardicin\_A\_biosynthetic\_gene\_cluster | 33 | 75.9219088937 | 189 | 7.8e-48 |
| AEI58882.1 | phenylglycolate\_oxidase | BGC0000455 | Vancomycin\_biosynthetic\_gene\_cluster | 36 | 75.4880694143 | 184 | 2.5e-46 |
| CAG15041.1 | HmO\_protein | BGC0000441 | Teicoplanin\_biosynthetic\_gene\_cluster | 35 | 75.9219088937 | 182 | 9.6e-46 |
| CAE53379.1 | Hmo\_protein | BGC0000440 | Teicoplanin\_biosynthetic\_gene\_cluster | 35 | 75.9219088937 | 182 | 9.6e-46 |
| AAG29798.1 | dehydrogenase | BGC0000833 | Coumermycin\_A1\_biosynthetic\_gene\_cluster | 32 | 83.5140997831 | 181 | 2.8e-45 |
| CAH18566.1 | putative\_L-lactate\_dehydrogenase,\_TobD3 | BGC0000721 | Tobramycin\_biosynthetic\_gene\_cluster | 30 | 75.704989154 | 163 | 6e-40 |
| EWS95127.1 | L-lactate\_oxidase | BGC0000306 | Arylomycin\_biosynthetic\_gene\_cluster | 40 | 35.1409978308 | 115 | 1.4e-25 |
| ALK27904.1 | putative\_hydroxyphenylglycine\_aminotransferase/hydroxymandelate\_oxidase\_fusion\_protein | BGC0001233 | Feglymycin\_biosynthetic\_gene\_cluster | 33 | 65.2928416486 | 111 | 2.7e-24 |
